# Direct and indirect costs attributable to musculoskeletal disorders in Belgium

**DOI:** 10.1093/eurpub/ckac130.049

**Published:** 2022-10-25

**Authors:** V Gorasso, J Van derHeyden, R De Pauw, I Pelgrims, K De Ridder, S Vandevijvere, S Vansteelandt, B Vaes, D De Smedt, B Devleesschauwer

**Affiliations:** 1 Epidemiology and Public Health, Sciensano, Brussels, Belgium; 2 Public Health and Primary Care, Ghent University, Ghent, Belgium; 3 Rehabilitation Sciences, Ghent University, Ghent, Belgium; 4 Chemical and Physical Health Risks, Sciensano, Brussels, Belgium; 5 Applied Mathematics and Statistics, Ghent University, Ghent, Belgium; 6 Medical Statistics, LSHTM, London, UK; 7 Public Health and Primary Care, KU Leuven, Leuven, Belgium; 8 Veterinary Public Health and Food Safety, Ghent University, Ghent, Belgium

## Abstract

**Background:**

Within the European Union, musculoskeletal (MSK) disorders represent the most prevalent and costly work-related health problems affecting about 45 million workers. Since middle-aged people during their formative and peak income-earning years are predominantly affected, MSK disorders are the major contributors to the loss of productive life years in the workforce compared with other non-communicable diseases. This study aimed to summarize the average yearly economic impact of low back pain (LBP), neck pain (NKP), osteoarthritis (OST) and rheumatoid arthritis (RHE) in Belgium from 2013 to 2017.

**Methods:**

Direct costs, measured by reimbursed expenditures for medical services and medications, were derived by the national health insurer. Indirect costs were computed by multiplying the mean number of days absent from work (derived by the Belgian health interview survey, as prevalence data) with the average gross daily wage. Multivariate regression models were used to explore the extent to which average yearly costs were associated with MSK disorders. The method of recycled predictions allowed to estimate the marginal effect of each MSK disorder on costs.

**Results:**

25% of Belgian adults were affected by at least one MSK disorder that incurred on average to 1,524€ per capita. LBP was the most costly disorder (2,405€ per capita) followed by NKP (2,260€ per capita). In the working population, 15% had at least one MSK disorder with an average indirect cost of 3,083€ per capita. People with LBP were the only showing a significantly higher indirect cost compared to a population without LBP, with an adjusted cost per capita of 5,875€.

**Conclusions:**

The adult Belgian population is largely affected by MSK disorders. Every year the total adjusted healthcare cost amounted to more than 3 billion Euros. Additionally, on average every year Belgium spends around 2 billion Euros for work absenteeism related to one of the MSK disorders.

**Key messages:**

- MSK disorders have a great societal cost in Belgium.
- Intervening on the working population that is largely affected can help reducing absenteeism costs.

